# A Simple Strain Typing Assay for *Trypanosoma cruzi*: Discrimination of Major Evolutionary Lineages from a Single Amplification Product

**DOI:** 10.1371/journal.pntd.0001777

**Published:** 2012-07-31

**Authors:** Raul O. Cosentino, Fernán Agüero

**Affiliations:** Instituto de Investigaciones Biotecnológicas, Universidad de San Martín, San Martín, Buenos Aires, Argentina; University of Pittsburgh, United States of America

## Abstract

**Background:**

*Trypanosoma cruzi* is the causative agent of Chagas' Disease. The parasite has a complex population structure, with six major evolutionary lineages, some of which have apparently resulted from ancestral hybridization events. Because there are important biological differences between these lineages, strain typing methods are essential to study the *T. cruzi* species. Currently, there are a number of typing methods available for T. cruzi, each with its own advantages and disadvantages. However, most of these methods are based on the amplification of a variable number of *loci*.

**Methodology/Principal Findings:**

We present a simple typing assay for *T. cruzi*, based on the amplification of a single polymorphic *locus*: the TcSC5D gene. When analyzing sequences from this gene (a putative lathosterol/episterol oxidase) we observed a number of interesting polymorphic sites, including 1 tetra-allelic, and a number of informative tri- and bi-allelic SNPs. Furthermore, some of these SNPs were located within the recognition sequences of two commercially available restriction enzymes. A double digestion with these enzymes generates a unique restriction pattern that allows a simple classification of strains in six major groups, corresponding to DTUs TcI–TcIV, the recently proposed Tcbat lineage, and TcV/TcVI (as a group). Direct sequencing of the amplicon allows the classification of strains into seven groups, including the six currently recognized evolutionary lineages, by analyzing only a few discriminant polymorphic sites.

**Conclusions/Significance:**

Based on these findings we propose a simple typing assay for *T. cruzi* that requires a single PCR amplification followed either by restriction fragment length polymorphism analysis, or direct sequencing. In the panel of strains tested, the sequencing-based method displays equivalent inter-lineage resolution to recent multi- *locus* sequence typing assays. Due to their simplicity and low cost, the proposed assays represent a good alternative to rapidly screen strain collections, providing the cornerstone for the development of robust typing strategies.

## Author Summary

*Trypanosoma cruzi*, the causative agent of Chagas Disease, infects approximately 8 million people in the Americas, with 200,000 new cases reported anually. The disease, in its chronic stage, has different manifestations: mega-colon, mega-esophagus, and cardiomyopathy, among others. The fact that infections by the same species cause these different clinical outcomes is believed to be determined, at least in part, by the genetic background of the parasite (infection by different strains). By analyzing a number of molecular markers, the population of the parasite has been divided into seven major evolutionary lineages, which evolve mostly independently, by clonal expansion with infrequent exchange of genetic material. Accurate classification of different strains and isolates into their corresponding evolutionary lineages is therefore essential to obtain a good map of biological, biochemical and ecoepidemiological features for the whole species. The current methods available to type *T. cruzi* stocks are either laborious and costly (requiring the amplification and sequencing of a variable number of genes or gene fragments), or limited in resolution. In this work we describe a number of key discriminant sites in a gene encoding a putative enzyme from the sterol pathway of the parasite, which were exploited to design a couple of alternative typing assays. Using these key discriminant sites, we can classify any *T. cruzi* stock into either six or seven evolutionary lineages using only one gene fragment, and in a matter of hours (depending on the assay used). To our knowledge, the proposed assays are the first typing assays that can discriminate *T. cruzi* stocks with such speed and low cost.

## Introduction

*Trypanosoma cruzi*, a protozoan parasite of the order Kinetoplastida, is the causative agent of Chagas Disease, a neglected disease that is is endemic in South America, affecting 8 million people in Latin America [1]. The *T. cruzi* species displays a considerable genetic and phenotypic diversity [2], [3], which is likely to be the result of a predominantly clonal mode of evolution through large time spans [4], [5]. By using a number of experimental approaches the population has been divided in different evolutionary lineages. A number of protein and genetic markers have been used to classify strains in two [6], [7] or three major lineages [6], [8], [9]. Other experimental strategies, such as RAPD and multilocus isoenzyme electrophoresis (MLEE) support the distinction of six subdivisions [10]–[12] referred to as discrete typing units (DTUs), originally designated as DTUs I, IIa, IIb, IIc, IId, and IIe [11]. Recently, this nomenclature was revised as follows: TcI, TcII (former TcIIb), TcIII (IIc), TcIV (TcIIa), TcV (TcIId) and TcVI (TcIIe) [13], [14], with a seventh DTU (Tcbat/TcVII) proposed around the same time [14], [15].

Lineages TcV and TcVI (which include the strain used for the first genomic sequence of *T. cruzi*, CL Brener) have a very high degree of heterozygosity but otherwise very homogeneous population structures with low intra-lineage diversity [16], [17]. The currently favored hypothesis suggest that these two lineages originated after either one or two independent hybridization events between strains of DTUs TcII and TcIII [17]–[19].

The biological variability displayed by *T. cruzi* has been correlated with its genetic diversity [20], [21]. Moreover, the clinical manifestations of Chagas Disease are also diverse, and have been suggested to be, at least in part, due to the genetic diversity of the parasite [22]–[25]. Based on these premises, a panel of experts have recommended the appropriate typing and naming of *T. cruzi* strains and isolates used in molecular, biochemical, and eco-epidemiological studies [13], [14].

As mentioned, there are already a number of typing methods available, based on different methodologies. After a revision of all diagnostic molecular characters described, Lewis *et al.* recommended a number of assays for rapid and accurate discrimination of all lineages [26]. However, even the most streamlined strategies require the analysis of a number of *loci* to discriminate strains from all currently recognized lineages. Currently the most widely used methods for typing *T. cruzi* strains and lineages are based on size polymorphisms of the spliced leader DNA gene (mini-exon) [7], [27], the 24S *α*-rRNA gene, and the 18S rRNA gene [12]. Other methods offer higher resolution (e.g. amplification of genomic DNA using random primers), but involve analysis of complex electrophoretic patterns [28]. These methods are theoretically able to unequivocally discriminate all lineages, but interpretation of results is often prone to errors because the discriminating power of the assay relies on the visual inspection of small differences in electrophoretic bands (e.g. 175 bp vs 165 bp vs 155 bp in the amplification product for the 18S rRNA gene [12]), or even the assessment of the intensities of some amplification products (e.g. the identification of a “weak” 125 bp as the basis for classifying a strain under lineage TcIId [12]).

Recently, a number of multi-locus sequence typing (MLST) markers were introduced for *T. cruzi*, bringing robust resolution of evolutionary lineages through sequencing of 4–10 diagnostic *loci* [16], [29]. These MLST assays provide a high discriminatory power, including the resolution of unique genotypes within each major DTU. Although perfectly fit for in-depth diversity studies, these assays are not simple enough for fast screening of large collections of isolates, or their implementation in the field.

Based on the identification of a number of discriminating polymorphisms in the gene encoding a putative C-5 sterol desaturase in *T. cruzi* we propose a simple typing strategy that relies on the amplification of a single *locus*, therefore providing a rapid assay that can be used routinely for typing *T. cruzi* strain collections.

## Methods

### *T. cruzi* stocks

Thirty three parasite stocks were analyzed, covering all major *T. cruzi* evolutionary lineages/DTUs, including *T. cruzi* reference strains, as well as isolates from bats that correspond to a new lineage/DTU [15], [30], and another bat trypanosome, *T. cruzi marinkellei*. Their names and corresponding lineage/DTU classification) are: Sylvio X10 cl1, Dm28c, TeDa2 cl4, PALV2-2cl5, CAI72, JR cl4, LL015, and Tul0 (TcI); MAS1 cl1, TU18 cl93, IVV cl4, and Y (TcII); M6241 cl6, M5631 cl5, Tc863, and X109/2 (TcIII); CanIII cl1, Dog Theis, Fuscicolis 15, and 92122102R (TcIV); Sc43 cl9, MN cl2, LL014, Teh53 cl4, and Bertha (TcV); Tulahuen cl2, CL-Brener, P63 cl1, and Tul2 (TcVI); TCC1994, and TCC1122 (Tcbat, code numbers are from the Trypanosomatid Culture Collection, Dept of Parasitology University of Sao Paulo); B3, and B7 (*T. cruzi marinkellei*). Genomic DNA from these strains was obtained from the T. cruzi collection at IIB-UNSAM, or were generous gifts from Dr. Patricio Diosque (Universidad de Salta, Argentina) and Dr. Marta M.G. Teixeira (Universidade de Sao Paulo, Brazil). The typing and lineage/DTU classification of these strains was carried out using the method proposed by S. Brisse, J Verhoef and M. Tibayrenc M [12] and by an implementation of the MLST assay [29] based on 4 *loci* (P. Diosque, personal communication).

### Oligonucleotides, PCR amplification, and sequencing

A fragment of 832 bp of the *TcSC5D* gene (CL-Brener genome *loci*: TcCLB.473111.10, TcCLB.507853.10) was amplified using the following oligonucleotide primers: *TcSC5D*-fwd 5′-GGACGTGGCGTTTGATTTAT-3′, *TcSC5D*-rev 5′-TCCCATCTTCTTCGTTGACT-3′. A fragment of 537 bp of the *TcMK* gene (CL-Brener genome *loci*: TcCLB.436521.9, TcCLB.509237.10) was amplified using the following oligonucleotide primers: Tc-Mev-kinase26-Fw 5′-TTTTTGCATGTCATTTTGG-3′, Tc-Mev-kinase662-Rv 5′-AGCGGTCTTGTAATGAGCAC-3′. PCR amplification was performed using Taq polymerase (Invitrogen) in a Biometra T Professional Gradient 96 cycler. Amplification mixtures contained 10 pmol of each primer, PCR buffer (Invitrogen), 1.6 mM MgCl_2_, 100 ng of genomic DNA, 200 µM dNTPs, 2.5 U Taq polymerase (Invitrogen), and water to a final volume of 25 µl. After denaturing at 94 for 4.5 min, thermal cycling was performed for 35 cycles at 94 for 30 s, followed by 30 s at 58, followed by 72 for 30 s. Reactions were finished by a 5 min incubation at 72. Amplification products were checked in 1.2% agarose gels stained with ethidium bromide. A single amplification product was observed for the TcSC5D product in all *T. cruzi* stocks tested, including those from the Tcbat lineage (not shown). The same oligonucleotide primers were used to amplify the corresponding gene fragment from *Trypanosoma rangeli* and *Trypanosoma cruzi marinkellei*. In these cases, the yield of the amplification was lower, but a product of the expected size could be amplified in all cases, although in *T. rangeli* a couple of additional, non-specific bands of smaller size (∼300–250 bp) were also amplified. For the TcMK gene, a single amplification product was obtained in all strains tested (except for those from the TcI DTU, due to the presence of polymorphisms in the 3′ end of the fwd primer. For direct sequencing of the amplification products, an aliquot (10 µl) of the amplification reaction was treated with 1 U of Exonuclease I (Fermentas) and 10 U of Shrimp Alkaline Phosphatase (Fermentas) for 45 mins at 37 and then for 30 min at 80 to inactivate these enzymes. Subsequently two sequencing reactions were prepared (fwd, rev), with the same primers used for the amplification of the product. Sequencing was carried out in an Applied Biosystems 3130 capillary sequencer using a Big-Dye terminator cycle sequencing kit, according to the instructions of the manufacturer. After sequencing, the length of each amplification product was such that, upon assembling a contig using the corresponding forward and reverse reads, there is a significant overlap, therefore providing sequence information from both DNA strands covering all key polymorphic sites.

### Sequence assembly and calling of heterozygous SNPs

Base calling of chromatograms, assembly of sequences, detection of polymorphisms (including heterozygous peaks) and manual inspection of assembled sequences and polymorphisms was done using a software package composed of Phred (version 0.020425.c) [31], [32], Phrap (version 0.9909329), Polyphred (version 5.04) [33] and Consed (version 15.0) [34]. Basecalling of chromatograms was done by Phred. Sequences were then assembled by Phrap. Polyphred was used to process phrap assemblies to detect polymorphic sites. All candidate SNPs identified by PolyPhred (score >70/99), including heterozygous peaks, were visualized with Consed.

### Restriction fragment length polymorphism analysis

All restriction enzymes were purchased from New England Biolabs (Ipswich, MA, USA). An aliquot (20 µl) of the *TcSC5D* amplification products where digested in a single incubation with 1 U of *Hpa*I (NEB R0105) and 1 U *Sph*I (NEB R0182) at 37 for 1 h. Digestion was performed in the same buffer used for PCR amplification (Invitrogen Taq PCR Buffer). The resulting restriction fragments were resolved by electrophoresis of in 2% TBE-agarose gels, applying 5,33 V/cm for 1 h. For *TcMK* fragment, an aliquot (20 µl) of the amplification products where digested in a single incubation with 1 U of *Xho*I (NEB R0146) at 37 for 1 h. The resulting restriction fragments were resolved by electrophoresis in 2.5% TBE-agarose gels, applying 5,33 V/cm for 1.5 h. The gels were stained with ethidium bromide for visualization under UV light.

### Data deposition

Sequences of the *TcSC5D* gene have been deposited in the GenBank database with the following accession numbers: HQ586986 (CL-Brener), and JN050564–JN050587 (batch submission for all other strains). Sequences of the *TcMK* gene have been deposited under the following accession numbers: HQ586987 (CL-Brener), and JN050693–JN050720. Heterozygous sequence polymorphisms have been submitted as ambiguities in the sequence using standard IUPAC notation (see Supplementary Figure S1). All polymorphic sites identified will also be made available in the next release of the TcSNP database [35].

### Locus identifiers

According to recent changes related to standardization in trypanosomatid *locus* identifiers used in community databases, all such *T. cruzi* identifiers referenced in this work appear in their current shorter form, e.g. TcCLB.507853.10 (equivalent to the old *locus* identifier Tc00.1047053507853.10).

## Results

### The highly polymorphic *TcSC5D* gene enables a simple lineage typing assay for *T. cruzi*

**Figure 1:**
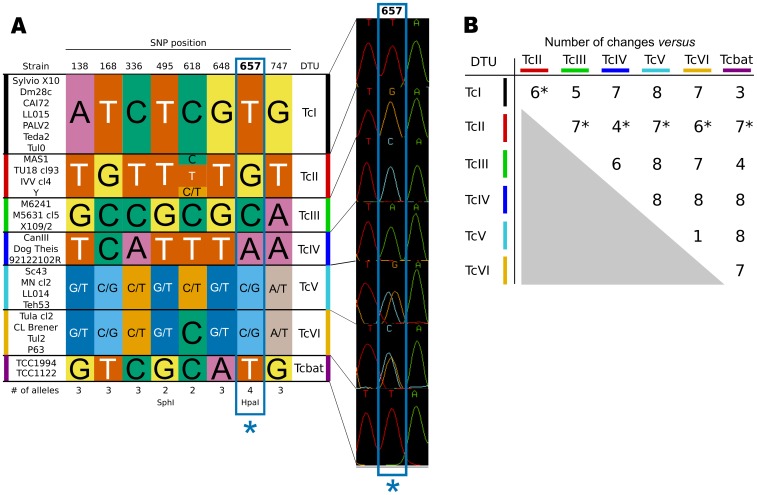
Key polymorphic sites in the *T. cruzi TcSC5D* gene. **A:** diploid genotypes built from identified highly informative sites (see main text), showing a section of one chromatogram around the tetra-allelic SNP at position 657. **B:** Summary of observed nucleotide changes between DTUs for these six sites. In the case of the comparisons with the TcII DTU, this is the minimum expected number of changes (marked with ^*^ in the figure), because of the diversity observed at position 618 in strains from this DTU.

When analyzing the genetic diversity in genes from the sterol biosynthesis pathway of *T. cruzi*, we turned our attention to the gene encoding a putative C-5 sterol desaturase (lathosterol/episterol oxidase). This gene, present as a single copy per haploid genome, and mapped to chromosome 22 [36], showed the highest density of fixed differences in this dataset (11.37 SNPs per 100 bp, Cosentino RO, and Agüero F, manuscript in preparation). When analyzing the diversity in the *TcSC5D* gene, we observed a total of 81 fixed differences (70 synonymous, 11 non-synonymous), the majority of which were bi-allelic (changing one specific nucleotide base for another). However, we also noticed the presence of some tri-allelic sites (a base is replaced by two different bases in the population), and 1 position where all possibilities (A, C, G, and T) were observed in the population. This tetra-allelic SNP alone, at position 657 of the *TcSC5D* coding sequence, produced a separation of *T. cruzi* strains in five groups, that matched their classification in discrete typing units (DTUs), as obtained by other methods (see Figure 1): DTU I (“T” in homozygosity), DTU II (“G” in homozygosity), DTU III (“C” in homozygosity), DTU IV (“A” in homozygosity), and a fifth group consisting of DTUs V and VI (“C” and “G” in heterozygosity). To validate these findings, we re-sequenced the *TcSC5D* gene in an expanded panel of 31 *T. cruzi* strains from different lineages (see Figure 1A), confirming the genotype observed for this site in all these strains. DNA from these strains was originally typed using the method proposed by Brisse *et al.* [12], and by an earlier implementation of the MLST method of Lauthier *et al.* (see Methods).

Because the sequence reads were derived from direct sequencing of amplification products, they contain information from both alleles in the form of heterozygous peaks in the chromatograms. Therefore, and in line with the recent work on MLST typing strategies [16], [29] we called them ‘diploid sequence types’ or DSTs. However, a phylogenetic reconstruction using these DSTs does not provide a clear separation of lineages/DTUs, due to the presence of a number of homoplasies in the dataset. An alignment of representative sequences obtained after re-sequencing of this *locus* is available in supplementary Figure S1, with candidate homoplasic traits marked in red boxes. After careful analysis of these re-sequencing data, we identified 8 key polymorphic sites (marked in blue boxes in Figure S1) that are highly informative with respect to the current lineage assignment for the panel of strains analyzed. These sites include i) the tetra-allelic site at position 657; ii) five tri-allelic sites at positions 138 (A, G, and T are the observed characters in the population), 168 (C, G, T), 336 (A, C, T), 648 (A, G, T), and 747 (A, G, T); and iii) two bi-allelic sites at positions 495 (G, T), and 618 (C, T) (Figure 1). Additional informative sites (bi-allelic, tri-allelic) are present in the dataset, including some that overlap the recognition sequences for commercially available restriction enzymes (discussed below).

By taking advantage of these polymorphic sites we devised a number of simple and complementary lineage typing assays that only require a single amplification (PCR) step to discriminate all major evolutionary lineages/DTUs.

### A sequencing-based typing assay

Using the information on polymorphic sites in the *TcSC5D* gene, an initial simple typing assay can be proposed that is based on the direct sequencing of the amplification product. By analyzing the chromatogram peaks of only three positions of the *TcSC5D* sequence (618, 648 and 657) it is possible to discriminate strains from all evolutionary lineages (see Figure 1). However, the robustness of the assay can be increased by inspecting additional discriminant positions. In Figure 1, the eight positions described above, corresponding to the most informative sites identified, were used to build the corresponding discriminating diploid sequence types. Using these sites, it is possible to correctly infer the ancestral lineage of a given *T. cruzi* stock by reconstructing the diploid genotype for these sequence positions. The classification of most strains/isolates is robust, and involves the observation of a minimum of 3 (out of 8) nucleotide changes between the TcI and Tcbat lineages, a minimum of 4 changes between the TcII vs TcIV and TcIII vs Tcbat haplotypes, and a minimum of 5–8 changes for the remaining lineage comparisons (see Figure 1B). Additional bi-allelic discriminant sites are available and can be included, increasing the number of changes that support each DTU classification (see Supplementary Figure S2). However, the worst case scenario is the discrimination of strains from lineages TcV and TcVI. For this comparison, only one discriminating site is available (the bi-allelic SNP at position 618), and the difference observed at this site is a loss of heterozygosity in lineage TcVI.

As proposed, the assay requires an accurate call of heterozygous peaks for hybrid strains, something that is easily done with free software packages, such as PolyPhred [33] (free for academic use) or VarDetect [37]. However, if necessary to avoid the calling of heterozygous (double) peaks, this procedure can be substituted by a more laborious strategy of cloning and sequencing a number of amplification products. This diploid sequence typing (DST) strategy is essentially similar to the one recently used by Yeo et al. [16], and Lauthier *et al.* [29], but applied in our case to only one *locus*.

### A restriction fragment length polymorphism assay based on the *TcSC5D* gene

**Figure 2:**
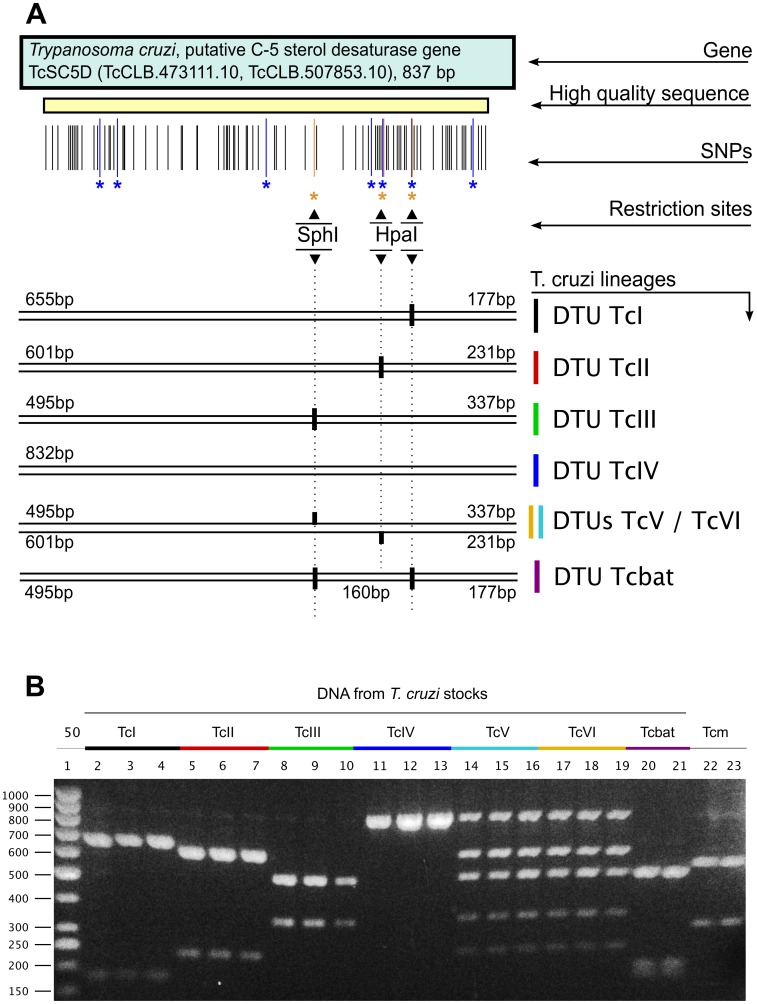
The TcSC5D locus as a lineage discriminant marker. **A:** schematic view of the TcSC5D amplicon, with all identified SNPs, including key discriminant positions (marked in blue), and polymorphic HpaI/SphI restriction enzyme sites, showing their presence/absence in each lineage. **B:** Restriction fragment length polymorphism analysis of the *TcSC5D* amplification product. Fragments of the SphI/HpaI double digestion were resolved in a 2% TBE-agarose gel. Lanes in the gel correspond to: molecular size markers (lane 1), and DNA from *T. cruzi* strains (lanes 2–21). These are: Sylvio X10 (lane 3), Dm28c (4), and CAI72 (5) for DTU TcI; MAS1 cl1 (6), TU18 cl93 (7), and IVV cl4 (8) for DTU TcII; M6241 cl6 (9), M5631 cl5 (10), and X109/2 (11) for DUT TcIII; CanIII cl1(12), Dog Theis (13) and 92122102R (14) for DTU TcIV; Sc43 (15), MN cl2 (16) and Teh53 (17) for DTU TcV; CL-Brener (18), P63 cl1 (19), and Tulahuen cl2 (20) for DTU TcVI; and TCC1994 and TCC1122 for DTU Tcbat. The corresponding locus from *T. cruzi marinkellei* was analyzed in lanes 22/23 (stocks B3 and B7).

When analyzing the sequence of the *T. cruzi TcSC5D* gene, we noticed the presence of polymorphic sites located within recognition sites for two commercially available restriction enzymes: *Sph*I (recognition site: GCATG∧C, isoschizomers BbuI, PaeI, and SpaHI), and *Hpa*I (recognition site: GTT∧AAC, isoschizomers BstHPI, and KspAI). Three such sites were identified in our re-sequencing study, and are listed in Table 1. An additional HpaI site (absent from all other *T. cruzi* sequences) was identified in *T. cruzi marinkellei*. These restriction enzyme sites are present alone and in homozygosity in three lineages/DTUs (TcI, TcII, TcIII), while one DTU (TcIV) does not contain any of these restriction sites, and the Tcbat lineage contains two of these restriction sites in homozygosity. Therefore a unique restriction pattern was expected to arise from a double digestion of the *TcSC5D* amplification product in strains from these DTUs. The two hybrid lineages (TcV and TcVI) contain two of these sites (see Table 1), both in heterozygosity, therefore we expected a pattern that is the logical addition of the patterns of lineages TcII and TcIII. A schematic view of the *TcSC5D* gene, showing all identified SNPs, and the mentioned restriction sites is shown in Figure 2A. And the result of the double digestion with these enzymes is shown in Figure 2B. In the interest of clarity, the restriction pattern is shown for three reference strains per DTU in the Figure, but the expected restriction pattern was observed in all (33) strains tested (not shown). Figure S1 contains a more detailed view of the gene, showing all available sequences aligned with the polymorphic restriction sites labeled.

**Table 1:** List of *Sph*I and *Hpa*I restriction enzyme sites in the *TcSC5D* gene.

DTU	SphI (497)	HpaI (site 1, 297)	HpaI (site 2, 604)	HpaI (site 3, 657)	Expected size(s) (bp)
TcI	NO	NO	NO	YES	655, 177
TcII	NO	NO	YES	NO	601, 231
TcIII	YES	NO	NO	NO	495, 337
TcIV	NO	NO	NO	NO	832
TcV	YES/NO	NO	YES/NO	NO	601, 495, 337, 231
TcVI	YES/NO	NO	YES/NO	NO	601, 495, 337, 231
Tcbat	YES	NO	NO	YES	495, 177, 160
Tcmk	NO	YES	NO	NO	541, 297

The location where the enzyme cuts the DNA is shown in parentheses. Coordinates are given with respect to the translational start codon (+1). The presence/absence of these sites in each DTU is noted in the table, as well as the expected number and sizes of fragments produced in a double digestion with these enzymes. All sites for lineages TcI-IV and Tcbat are present or absent in homozygosity. Some sites for lineages TcV and TcVI are present in heterozygosity, and are annotated as YES/NO, indicating both presence and absence of the site. Tcmk = *T. cruzi marinkellei*.

By taking advantage of these polymorphic sites, we propose a second alternative typing assay based on the same *locus* that involves the digestion of the TcSC5D amplicon with a mixture of SphI and HpaI restriction enzymes, followed by the separation of the resulting restriction fragments by gel electrophoresis, as shown in Figure 2. Because the two enzymes are optimally active in buffers of similar composition (NEB Buffer 4), only a single incubation step is required to perform a double digestion. Moreover, the enzymes are also highly active in Taq polymerase (PCR) buffer, so the double digestion can be performed right after the amplification step, in the same tube, therefore enabling a highly streamlined protocol for strain typing.

As can be seen in the agarose gel electrophoresis, the digestion with a mixture of these enzymes generates 7 unique restriction patterns (if one also considers the pattern observed for *T. cruzi marinkellei*). These patterns are unique for all DTUs, except for DTUs TcV and TcVI, that cannot be differentiated. In the case of these DTUs, the banding pattern is essentially the sum of the patterns of DTUs TcII and TcIII, which agrees with the currently favored hybridization hypothesis [18]. In this pattern, apart from the theoretically expected fragments, the hybrid stocks (DTUs TcV and TcVI) show an additional 832 bp band. This size corresponds to the size of the undigested amplification product, and is most probably the result of heteroduplex formation during the PCR amplification step. This artifact has been described already, and is due to the presence of closely related amplification products in the mixture [38], [39]. The chimeric double strand DNA molecules are produced by the repeated melting and re-annealing during PCR, which result in increased formation of these heteroduplexes at the final amplification cycles [38]–[40].

For comparison purposes, we analyzed DNA from the related bat trypanosome, *Trypanosoma cruzi marinkellei* (already discussed above), and also from the co-endemic trypanosome *Trypanosoma rangeli*. As shown, the RFLP analysis of the *T. cruzi marinkellei* genes produced a unique pattern of digestion that was different from those observed for *T. cruzi* DTUs (see Figure 2, lanes 22–23), including the recent Tcbat DTU/lineage. This was caused by an additional HpaI site, created by the accumulation of 4 nucleotide changes around position 292–298 in this lineage (see Figure S1). For *T. rangeli*, although the analysis in this case is hampered by the presence of spurious amplification bands (see Methods), the comparison of untreated and digested samples revealed a restriction pattern that appears to be similar to that observed for *T. cruzi* DTU TcI (not shown).

### A second PCR-RFLP assay discriminates DTUs TcV and TcVI

**Figure 3:**
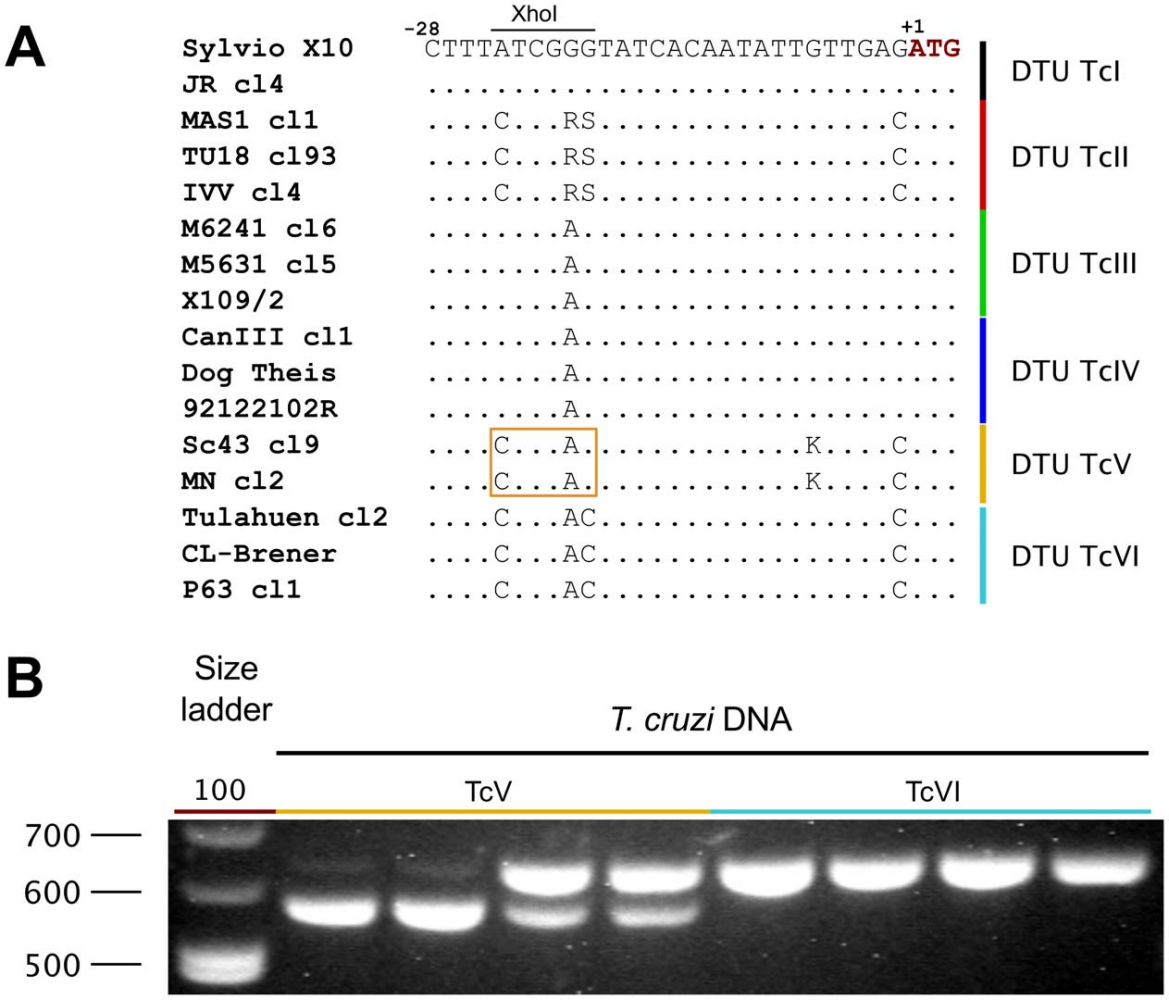
Restriction fragment length polymorphism analysis of the *TcMK* amplification product. **A:** a multiple sequence alignment of the 5′-UTR just upstream of the translational start codon, showing the polymorphic *Xho*I site. A solid line box marks the *Xho*I site in TcV strains. **B:** Agarose gel electrophoresis showing a PCR-RFLP analysis of selected strains. Strains analyzed were: Sc43 cl9, MN cl2, LL014 and Teh53 for DTU TcV; and Tulahuen cl2, CL-Brener, Tul2 and P63 cl1 for DTU TcVI.

Although discrimination of DTUs TcV and TcVI can be attained by sequencing of the *TcSC5D* amplification product, in some cases a PCR-RFLP assay is desirable (e.g. to perform strain typing assays in the field or in remote settings where access to Sanger sequencing technology is limited). By including a second *locus* in the assay, it is possible to discriminate DTUs TcV from TcVI using a fast PCR-RFLP assay. In our analysis of the sterol biosynthesis pathway (Cosentino R and Agüero F, manuscript in preparation), we noticed another discriminating region in the mevalonate kinase gene (*TcMK*, CL-Brener *loci*: TcCLB.436521.9, TcCLB.509237.10), overlapping a *Xho*I site (recognition site: C∧TCGAG, isoschizomer PaeR7I). Within the 5′-UTR of this gene (at position −24 from the translational start codon), we identified a conserved *Xho*I site in strains from the TcV DTU (in some cases in heterozygosity), that is absent altogether in strains from the TcVI DTU (see Figure 3 A). Therefore, a digestion of the 637 bp amplification fragment produces the restriction pattern shown in Figure 3B. As can be observed, strains from the TcV DTU all show the 575 bp band corresponding to the expected digestion product, whereas TcVI strains (and the heterozygous TcV strains) show a band of 637 bp corresponding to the undigested product.

## Discussion

In this work we propose a simple and fast typing assay for *T. cruzi* that can classify a *T. cruzi* strain/isolate into any of the seven major evolutionary lineages (TcI–TcVI, and Tcbat) by sequencing a single amplification product and focusing the analysis on just 8 discriminant polymorphic sites. A faster PCR-RFLP variant of the typing assay can generate seven unique restriction patterns, effectively classifying strains in six major *T. cruzi* groups, and is also able to discriminate isolates from a related species, such as *T. cruzi marinkellei*. The majority of these patterns have only 2 bands, and all are nicely resolved in 2% agarose gels, greatly facilitating interpretation of the typing assay.

**Figure 4:**
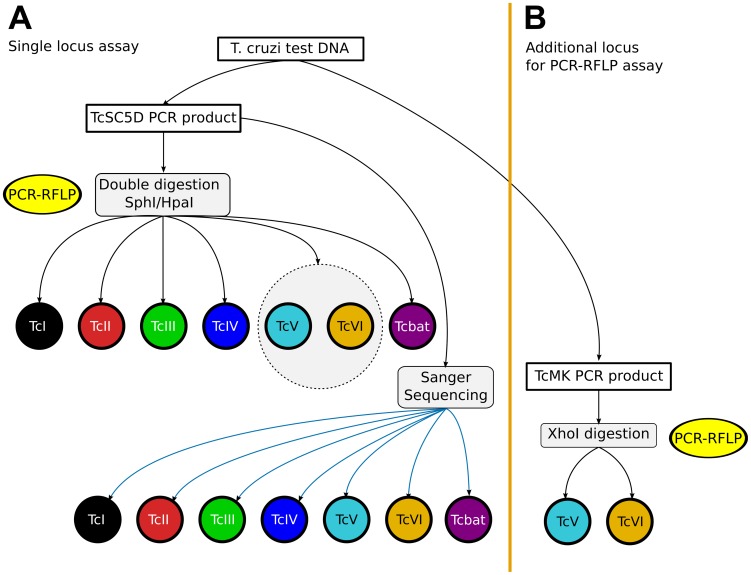
Proposed typing strategy based on the *TcSC5D* locus. **A**: the highly streamlined TcSC5D-PCR-RFLP assay can discriminate all non-hybrid lineages. If discrimination of DTUs TcV from TcVI is necessary, sequencing of the *TcSC5D* amplification locus is required. **B**: alternatively, if the method of choice is the PCR-RFLP, then a second *locus* can be assayed to resolve these DTUs. This additional *locus* can be the *TcMK* gene as shown in the figure (this work, see main text), or the gp72 gene (polymorphic *Taq*I site [41]).

Although this faster assay fails to discriminate DTUs TcV from TcVI, it can discriminate TcV/TcVI (as a group) from all other DTUs, and can therefore be used to rapidly screen a large collection of *T. cruzi* DNA samples. A schematic view of the proposed typing strategy is shown in Figure 4. In this strategy, we propose the use of a second *loci*, if resolution of lineages TcV and TcVI is required. In the figure, this marker is the polymorphic XhoI site in the TcMK gene (see Results). But this marker can be replaced by the single-copy gp72 gene (polymorphic TaqI site) [41] or other similar markers.

With some caveats, the PCR-RFLP assay can also be applied to detect mixed stocks (isolates with different parasite populations). We have applied the PCR-RFLP typing assay to artificial DNA mixtures and have found that the method behaves as theoretically expected. For this we have prepared artificial mixtures for each DTU, in which we contaminated the DNA with increasing quantities of DNA from another DTU. In all cases amplification of the TcSC5D gene fragment was not affected, and the restriction pattern obtained was the sum of the expected banding pattern of the DTUs present in each mixture, with the intensities of the bands serving as indicators of the relative level of contamination (not shown). This essentially means that when dealing with isolates containing parasites from different lineages, the assay can help identify a mixed isolate (with one exception). The exception is a theoretical isolate that is a mixture of parasites from lineages TcII and TcIII. If the isolate has a 50∶50 mixture of strains, then this mixture will be indistinguishable from a TcV/TcVI hybrid strain. Therefore we believe the method has also great potential to rapidly screen field isolates, and detect candidate cases for cloning of parasite stocks.

Many strain typing assays have been devised for *T. cruzi*, and so it may seem redundant to propose yet another molecular marker for this purpose. However, there is still an unmet need for a simple and low cost typing analysis that can be employed in clinical and/or eco-epidemiological samples. Also, as recognized recently by a number of experts, there is an increasing need for clearly typing and reporting the classification of *T. cruzi* strains in scientific communications [13], [14]. Finally, anecdotal (unpublished) data suggest that laboratory strain mixup is not rare (discussed in [42]). Availability of rapid typing methods can greatly improve the current situation by enabling fast typing of samples in many settings, and the routine typing of *T. cruzi* strains and isolates maintained in culture or as cryopreserved material. Because the proposed PCR-RFLP typing strategy is simple, and can produce results in hours, it can be also used as a preamble to more complex classification schemes.

The two proposed markers (TcSC5D and TcMK) are both single copy genes, that are apparently under purifying selection, with a ratio of non-synonymous to synonymous changes of 0.16 and 0.39, respectively (data not shown for the TcMK gene). The fact that the same oligonucleotide primers are able to amplify the expected fragment from all evolutionary lineages, and from other related species such as *T. cruzi marinkellei* and *T. rangeli* (although with lower yields in these cases, see Methods) suggest that these are stable markers, appropriate for use in typing assays.

### Advantages of the proposed methods

Because the proposed assay is dependent on the amplification of a single *locus*, its main advantage is the ability to rapidly screen a collection of DNA samples from different isolates. When using the highly streamlined RFLP assay, results can be obtained in one day in a matter of hours. To our knowledge this is the only currently available typing assay that can discriminate strains with such speed and low cost. This fast typing method can classify *T. cruzi* strains in 6 groups, corresponding to DTUs TcI, TcII, TcIII, TcIV, Tcbat, and DTUs TcV and TcVI (as a group) (see Figure 4). Alternatively, the single- *locus TcSC5D* amplification product can be submitted for Sanger sequencing to resolve the heterozygous polymorphic site at position 618, to resolve the TcV/TcVI genotypes. In this case, results can be obtained in 2–3 days, depending on the average turnaround time for sequencing at each institution.

As mentioned already, there are a number of alternative methods available to type *T. cruzi* strains and isolates. Because it does not depend on sequencing of amplification products, the typing strategy proposed by Brisse *et al.* [12] is highly popular in many laboratories. With this method results can be obtained relatively quickly after PCR amplification. However, the method requires the amplification of fragments from 3 *loci* for each strain/isolate, the assessment of small differences in size (∼15 bp) between amplification products, and the comparison of band intensities. Furthermore, all this information has to be integrated into a complex decision tree to classify each sample (see Figure 2 in [12]), increasing the difficulty of the assay.

More recently, robust typing strategies have been proposed for use in eco-epidemiological and evolutionary studies [16], [29]. However, these methods do not degrade gracefully for quick typing of a strain collection as they require the amplification and sequencing of at least 4 *loci*. Other typing assays based on a PCR-RFLP strategy have been proposed, but they all fall short in terms of inter-lineage discrimination [26]. For example, only by combining data from three PCR-RFLP assays (based on 3 *loci*/restriction enzyme combinations) it was possible to obtain unique restriction profiles for DTUs TcI-TcIV, still failing to discriminate TcV from TcVI [26].

The proposed assay strategy therefore, provides a key advantage over other currently available strategies, and that is its speed, based mostly on the fact that only one PCR reaction is needed for each sample, independently of the DTU/lineage. Furthermore, the post-amplification processing of samples is minimal, and is reduced to either an enzymatic cleanup incubation for Sanger sequencing, or an enzymatic digestion for the RFLP assay (see Methods). In either case, typing of a collection of clones or strains can be scaled up further by using multi-well plates for both the amplification and the cleanup or double digestion steps. Another benefit of the method is that the double digestion step can be performed right after the amplification step, in the same buffer (there is no requirement to further purify or manipulate the amplified DNA). Finally, the readout of the sequencing assay is very simple, as it is highly focused on key discriminant sites. In the case of the PCR-RFLP assay, the nice resolution of all bands greatly facilitates the comparison of the restriction patterns (see Figure 2).

### Caveat emptor

In spite of these advantages, it is important to emphasize a number of potential issues with this assay. First of all, the same feature that makes this strategy simple, cheap and rapid (e.g. being a single-locus assay) could easily turn into a major weakness, for example, if novel homoplasic traits are uncovered at any of the key discriminating sites when sampling additional strains. In the TcSC5D *locus* there are a number of homoplasic characters. Most notably, the key SNP at position 618 (selected because of its discrimination of DTUs TcV/TcVI) has one such trait in strains of lineage TcII. Also, when including additional strains in our panel, we uncovered an additional homoplasic trait in the tri-allelic SNP at position 138, in the case of the TcIV strain Fuscicolis 15. It has been shown already that the use of single molecular markers can lead to misclassification [12], [43], [44]. In case of doubt, we would recommend that the amplification products be sequenced, or that additional markers are used to confirm/reject the classifications. Also, the fact that the typing assay relies on the amplification of a single copy per haploid genome locus means that its sensitivity will suffer in comparison with other assays relying on repetitive markers [43].

Secondly, when using the Sanger based sequencing assay, the analysis must focus on key discriminant positions in the sequence. As mentioned, there are a number of putative homoplasies in the dataset that would affect the results if all polymorphic sites are considered in the analysis without further inspection. These putative homoplasic traits correspond to those polymorphic sites in the alignment where a strain deviates from the expected character state for its current lineage/DTU classification (see Figure S1). That means they are homoplasic (by definition) in the context of the currently accepted classification. It is certainly possible that some of these character states are shared by strains from different DTUs, therefore reflecting a truly shared ancestry. However, for the purposes of developing a typing assay, we decided to ignore these sites. The presence of these shared traits across different DTUs have been noticed by others [16], [17], [29], [45], and are the reason for a number of apparently incongruent phylogenetic trees. The solution in many cases has been to focus the analysis on discriminant sites [45] or to increase the number of *loci* (effectively incrementing the number of polymorphic sites analyzed) to gain discriminatory power [16], [17], [29].

As shown in the results, the discrimination of stocks from DTUs TcV and TcVI relies on a single discriminant site in the case of the TcSC5D gene. In the case of the TcMK gene, there are another 6 discriminant sites that separate strains from these DTUs, all of which represent cases of loss of heterozygosity (not shown), that can be exposed by Sanger-based sequencing of the amplification product. However, only one of these allows a PCR-RFLP assay. In this regard it is also important to mention that in recent works analyzing a number of *loci*, these ‘loss of heterozygosity’ events in TcVI were observed only in some stocks, and only for some *loci* [16], [29], so further studies, using an expanded panel of strains are needed to validate these markers.

The observation of so few discriminant sites between DTUs TcV and TcVI is not surprising, given the relatively recent origin of these DTUs [17], and because the majority of polymorphic sites in sequences from these DTUs are expected to be in heterozygosity, and shared by alleles from the TcII and TcIII DTUs (their proposed parental types) [14], [18], [19].

Finally, although the RFLP assay is rapid and simple, the sequencing-based assay suffers from the same issues as the diploid sequence typing strategies recently published [16], [29]: namely the need for accurate calling of heterozygous peaks in chromatograms or its substitution by a more laborious cloning and sequencing strategy. As mentioned, the only difference identified between strains from DTUs TcV and TcVI in each gene is a loss of heterozygosity in one SNP. Because of this fact, there is a strict requirement to obtain good sequence quality around position 618 (*TcSC5D*) (or throughout the TcMK gene fragment) to increase the confidence in the base call at these sites.

### Conclusion

Based on the identification of key discriminant SNPs, and a number of polymorphic restriction sites, we propose two alternative simple typing strategies for *T. cruzi* that can rapidly classify strains into six or seven discrete typing units (DTUs). The method is easy to implement, cheap, and can produce results in a matter of hours or days, therefore lowering the barrier to routine typing of strains in the field and in research laboratories working with *T. cruzi*. To our knowledge this is the only currently available typing assay that can discriminate strains with such speed and low cost.

## Supporting Information

Figure S1 Multiple sequence alignment of *TcSC5D* sequences. Thirty three (33) sequences were obtained by re-sequencing of the *TcSC5D locus* in *T. cruzi* (different DTUs) and *T. cruzi marinkellei*. The actual sequence is shown only for the first strain (Sylvio X10). For the rest of the sequences, only nucleotides differing from the top sequence are shown. Putative homoplasic traits (enclosed in red boxes), correspond to those sites where a strain deviates from the expected character state for its current DTU/lineage classification. Key informative polymorphic sites (see Figure 1) are enclosed in blue boxes. Polymorphic restriction enzyme sites are also boxed, with solid lines (homozygous) or dotted lines (heterozygous). Ambiguities (heterozygous characters) are denoted using standard IUPAC notation.
(PDF)

Figure S2 Nucleotide changes observed between *T. cruzi* Discrete Typing Units. The table shows the total number of nucleotide changes observed between different DTUs. These include the 8 key informative changes shown in Figure 1, as well as other high-quality SNPs identified by re-sequencing.
(PDF)
