# Supplementary material for: A Simple Strain Typing Assay for Trypanosoma cruzi: Discrimination of Major Evolutionary Lineages from a Single Amplification Product
Source: PLoS Negl Trop Dis. 2012 Jul 31;6(7):e1777. doi: 10.1371/journal.pntd.0001777 (PMC3409129; doi:10.1371/journal.pntd.0001777)
Supplement: Figure S1 — Multiple sequence alignment of TcSC5D sequences. Thirty three (33) sequences were obtained by re-sequencing of the TcSC5D locus in T. cruzi (different DTUs) and T. cruzi marinkellei. The actual sequence is shown only for the first strain (Sylvio X10). For the rest of the sequences, only nucleotides differing from the top sequence are shown. Putative homoplasic traits (enclosed in red boxes), correspond to those sites where a strain deviates from the expected character state for its current DTU/lineage classification. Key informative polymorphic sites (see Figure 1) are enclosed in blue boxes. Polymorphic restriction enzyme sites are also boxed, with solid lines (homozygous) or dotted lines (heterozygous). Ambiguities (heterozygous characters) are denoted using standard IUPAC notation. (PDF) [file pntd.0001777.s001.pdf]

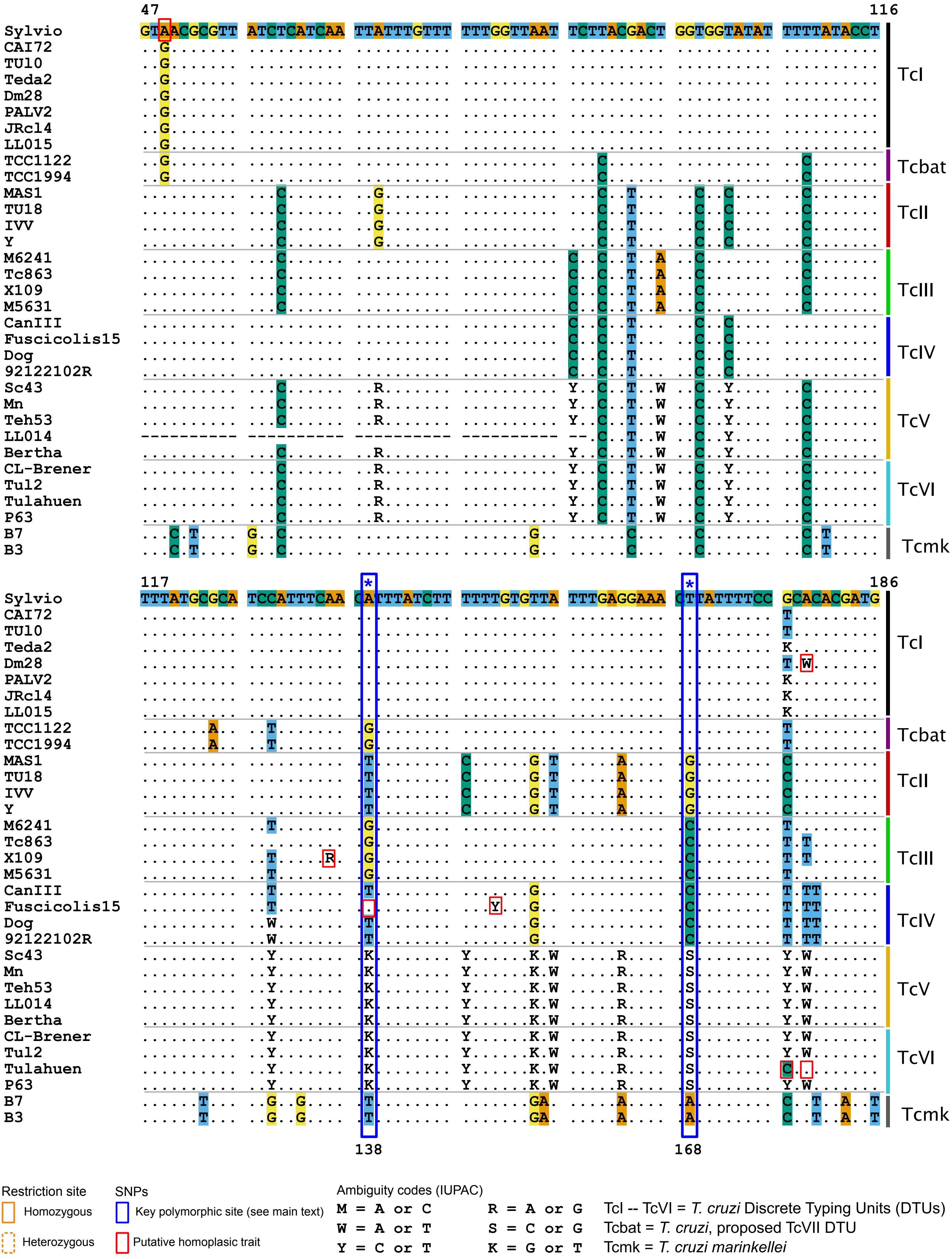
47
116
117
186
138
168
Sylvio
CAI72
TU10
Teda2
Dm28
PALV2
JRcl4
LL015
TCC1122
TCC1994
MAS1
TU18
IVV
Y
M6241
Tc863
X109
M5631
CanIII
Fuscicolis15
Dog
92122102R
Sc43
Mn
Teh53
LL014
Bertha
CL-Brener
Tul2
Tulahuen
P63
B7
B3
TcI
Tcbat
TcII
TcIII
TcIV
TcV
TcVI
Tcmk
Restriction site
Homozygous
Heterozygous
SNPs
Key polymorphic site (see main text)
Putative homoplasic trait
Ambiguity codes (IUPAC)
M = A or C
W = A or T
Y = C or T
R = A or G
S = C or G
K = G or T
TcI -- TcVI = *T. cruzi* Discrete Typing Units (DTUs)
Tcbat = *T. cruzi*, proposed TcVII DTU
Tcmk = *T. cruzi marinkellei*

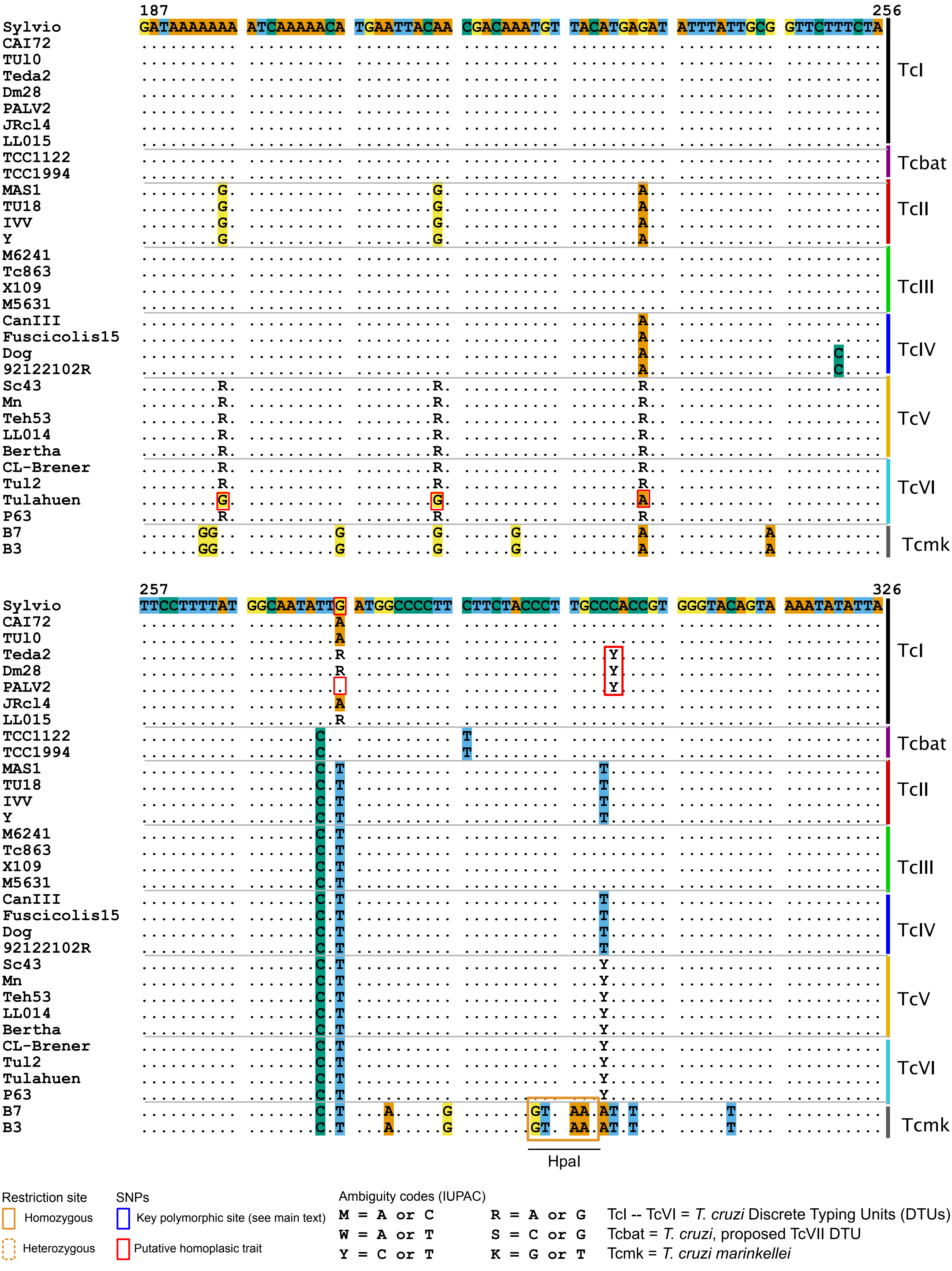
187
256
257
326
Sylvio
CAI72
TU10
Teda2
Dm28
PALV2
JRcl4
LL015
TCC1122
TCC1994
MAS1
TU18
IVV
Y
M6241
Tc863
X109
M5631
CanIII
Fuscicolis15
Dog
92122102R
Sc43
Mn
Teh53
LL014
Bertha
CL-Brener
Tul2
Tulahuen
P63
B7
B3
TcI
Tcbat
TcII
TcIII
TcIV
TcV
TcVI
Tcmk
HpaI
Restriction site
Homozygous
Heterozygous
SNPs
Key polymorphic site (see main text)
Putative homoplasic trait
Ambiguity codes (IUPAC)
M = A or C
W = A or T
Y = C or T
R = A or G
S = C or G
K = G or T
TcI -- TcVI = T. cruzi Discrete Typing Units (DTUs)
Tcbat = T. cruzi, proposed TcVII DTU
Tcmk = T. cruzi marinkellei

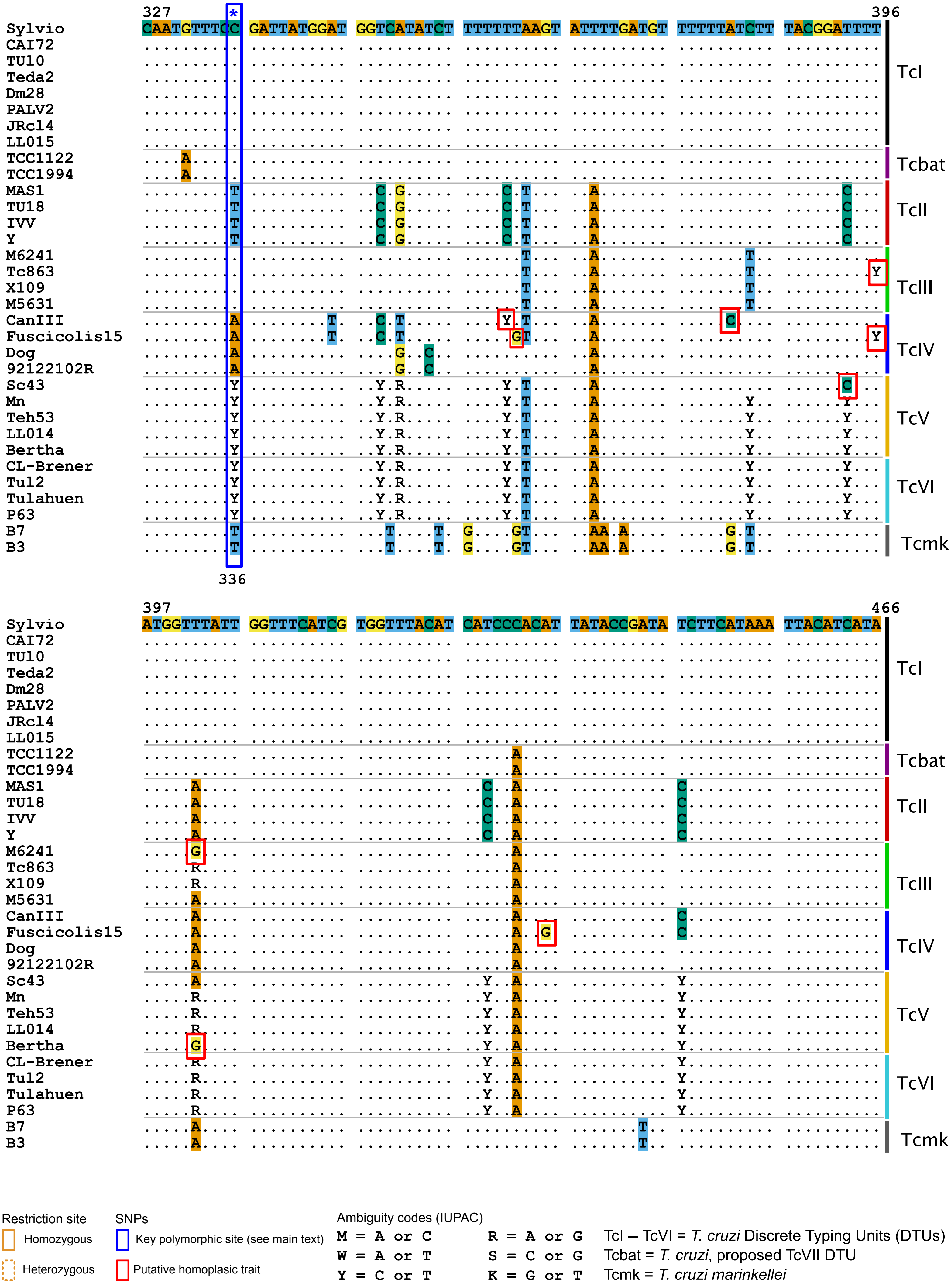
327
396
336
397
466
Sylvio
CAI72
TU10
Teda2
Dm28
PALV2
JRcl4
LL015
TCC1122
TCC1994
MAS1
TU18
IVV
Y
M6241
Tc863
X109
M5631
CanIII
Fuscicolis15
Dog
92122102R
Sc43
Mn
Teh53
LL014
Bertha
CL-Brener
Tul2
Tulahuen
P63
B7
B3
TcI
Tcbat
TcII
TcIII
TcIV
TcV
TcVI
Tcmk
Restriction site
Homozygous
Heterozygous
SNPs
Key polymorphic site (see main text)
Putative homoplasic trait
Ambiguity codes (IUPAC)
M = A or C
W = A or T
Y = C or T
R = A or G
S = C or G
K = G or T
TcI -- TcVI = T. cruzi Discrete Typing Units (DTUs)
Tcbat = T. cruzi, proposed TcVII DTU
Tcmk = T. cruzi marinkellei

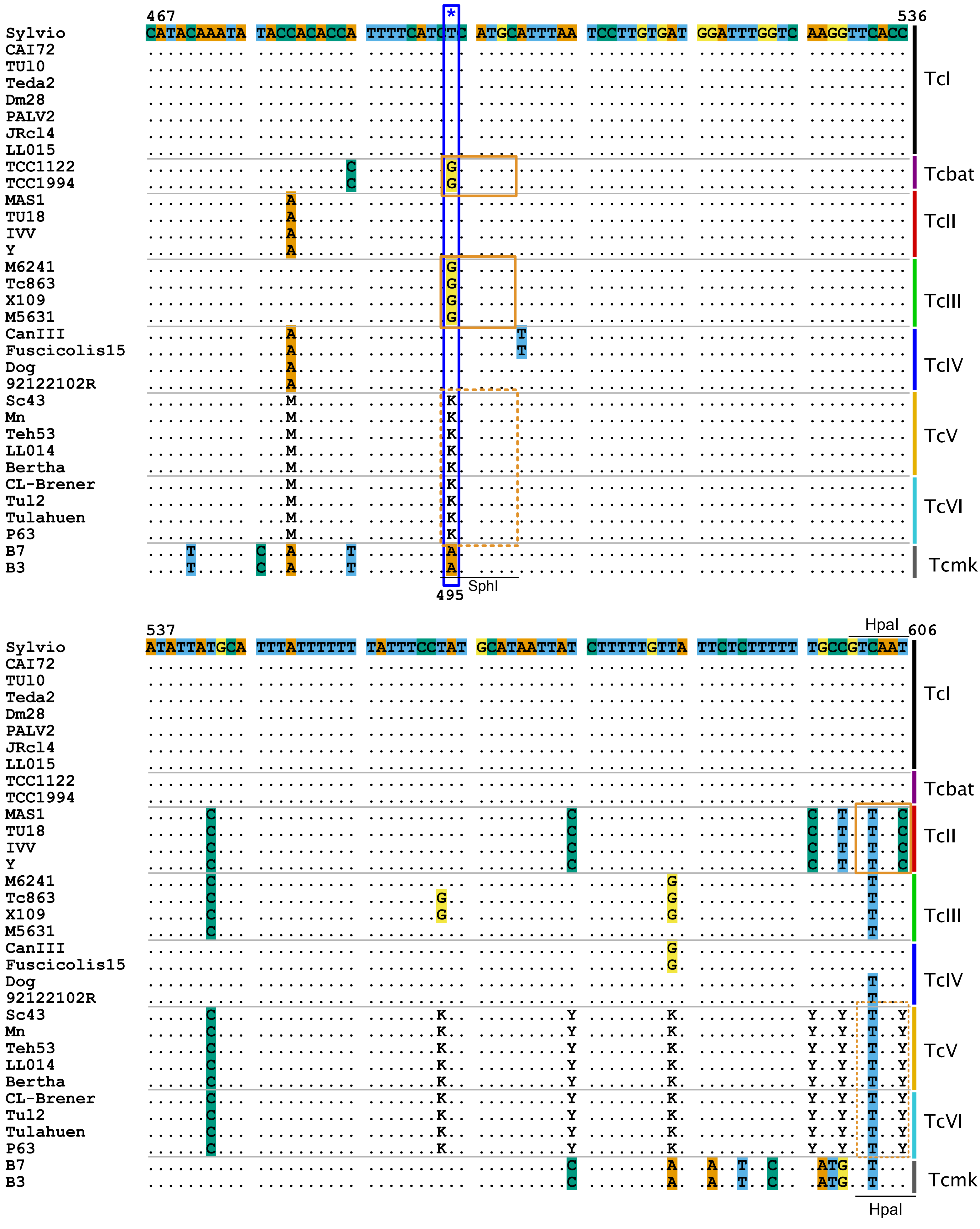


Restriction site
Homozygous
Heterozygous

SNPs
Key polymorphic site (see main text)
Putative homoplasic trait

Ambiguity codes (IUPAC)

M = A or C
W = A or T
Y = C or T
R = A or G
S = C or G
K = G or T

TcI -- TcVI = *T. cruzi* Discrete Typing Units (DTUs)
Tcbat = *T. cruzi*, proposed TcVII DTU
Tcmk = *T. cruzi marinkellei*

Seaview [blocks=10 fontsize=10 A4].

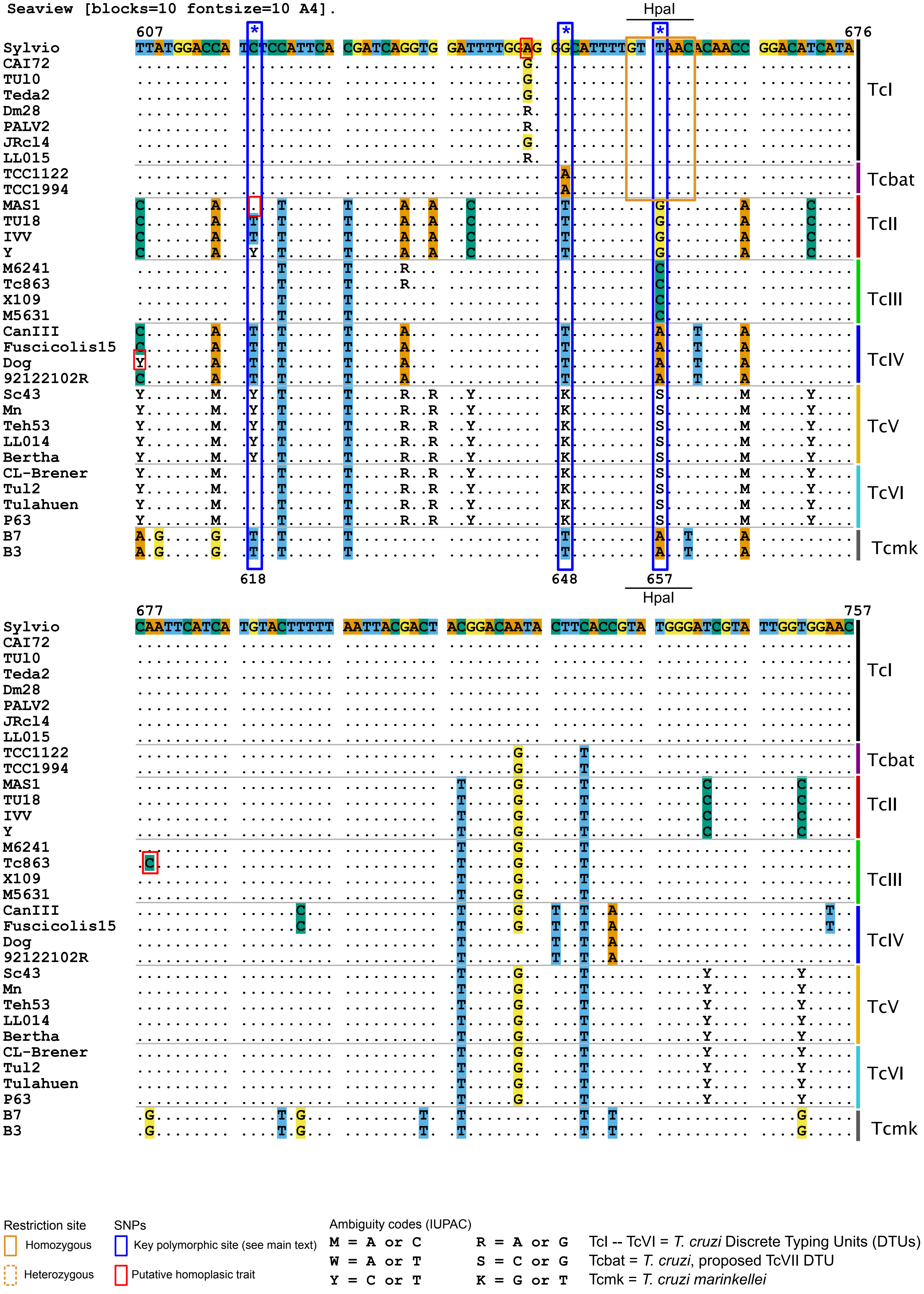

TcSC5D alignment. Cosentino RO & Agüero F (2012). Supplementary Figure S1. 
Seaview [blocks=10 fontsize=10 A4].

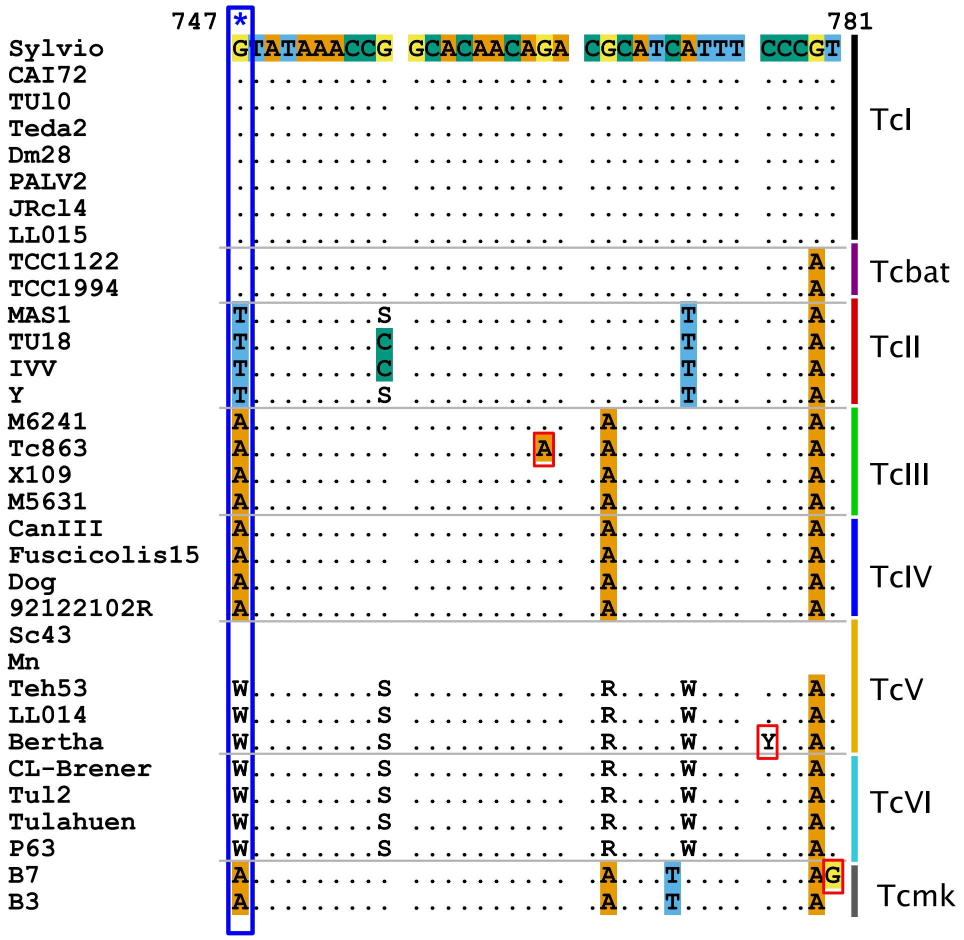


Restriction site
Homozygous
Heterozygous

SNPs
Key polymorphic site (see main text)
Putative homoplasic trait

Ambiguity codes (IUPAC)

M = A or C
W = A or T
Y = C or T
R = A or G
S = C or G
K = G or T

TcI -- TcVI = *T. cruzi* Discrete Typing Units (DTUs)
Tcbat = *T. cruzi*, proposed TcVII DTU
Tcmk = *T. cruzi marinkellei*
