# Supplementary material for: A Simple Strain Typing Assay for Trypanosoma cruzi: Discrimination of Major Evolutionary Lineages from a Single Amplification Product
Source: PLoS Negl Trop Dis. 2012 Jul 31;6(7):e1777. doi: 10.1371/journal.pntd.0001777 (PMC3409129; doi:10.1371/journal.pntd.0001777)
Supplement: Figure S2 — Nucleotide changes observed between T. cruzi Discrete Typing Units. The table shows the total number of nucleotide changes observed between different DTUs. These include the 8 key informative changes shown in Figure 1, as well as other high-quality SNPs identified by re-sequencing. (PDF) [file pntd.0001777.s002.pdf]

Nucleotide changes observed between DTUs for the TcSC5D locus.
Cosentino RO & Agüero F (2012). Supplementary Figure S2

Number of changes *versus*

DTU	TcII	TcIII	TcIV	TcV	TcVI	Tcbat
TcI	59	29	38	68	67	14
TcII		44	36	48	43	57
TcIII			25	48	46	24
TcIV				55	54	38
TcV					1	65
TcVI						63
